# Potential Hemoglobin A/F role in clinical Malaria

**DOI:** 10.6026/97320630013269

**Published:** 2017-08-31

**Authors:** Vikky Awasthi, Debprasad Chattopadhyay, Jyoti Das

**Affiliations:** 1 Immunology Division, ICMR-National Institute of Malaria Research, Dwarka, New Delhi; Present Address: Regional Medical Research Centre, Belagavi, Nehru Nagar, National Highway No.4, Belagavi-590010, Karnataka, India; 2 ICMR Virus Unit, Calcutta, ID & BG Hospital, GB 4, Beliaghata, Kolkata, India

**Keywords:** Hemoglobin, Plasmodium falciparum, Amino acids, Malaria

## Abstract

The Malarial parasite resides in the host RBC during its erythrocytic cycle. Plasmodium meets its entire nutritional requirement from RBC. It scavenges the hemoglobin of RBCs to meet its amino acid requirement. The host hemoglobin is made of different chains and it is dependent on age. Hemoglobin F (HbF), which has two-alpha and two gamma chain persists in children upto six years, and hemoglobin A (HbA) made of two alpha and two beta chains dominates. Therefore, it is of interest to compare the compositional features of HbA with HbF. Isoleucine is present in hemoglobin of children (gamma chain of HbF) while it is absent in adult hemoglobin (HbA). The presence of Isoleucine (I) makes HbF ideally suitable for the growth of parasite, as it does not have to depend upon the exogenous supply of the isoleucine, which might be responsible for making children more vulnerable to malaria as compared to adults.

## Background

Malaria is one of the most prevalent diseases in developing countries [1]. Malaria is caused by Plasmodium and over more than 200 species of Plasmodium has been identified. Four species of Plasmodium that causes malaria in humans are Plasmodium falciparum, Plasmodium vivax, Plasmodium malariae, Plasmodium ovale and recently, Plasmodium Knowleski have been reported to infect humans [2]. Malaria infection occurs when an infected mosquito bites a healthy host and injects sporozoites in blood stream. The sporozoite reaches liver and enters the hepatocytes, where it multiplies and forms merozoite, and these merozoites are released into the blood stream [3]. These merozoites infect the red blood cells (RBC). Parasite undergoes multiple division and morphological changes, which helps it to bypass immune invasions [4]. Plasmodium falciparum is responsible for deaths due to malaria [1]. Malaria parasite meets most of its nutritional requirements from blood. During replication hemoglobin is degraded to get amino acid required for growth [5]. Importance of amino acids on growth of Plasmodium has been as here discussed with reference to I (Isoleucine) [6], N (Asparagine) [7], M (methionine) [8] R (Arginine) [9] hibernation of Plasmodium [10]. Different Forms of hemoglobin's have been reported in the RBC according to human age [11]. Hemoglobin comprises of two alpha and two zetta or etta chains during embryonic stages when hematopoiesis starts in liver [12]. Later during the developmental stages hemoglobin having two alpha chains and two-gamma chain dominates. A significant amount of fetal hemoglobin persists upto eight months after birth. Most people have only trace amounts, if any, of fetal haemoglobin after infancy. The combination of two alpha genes and two beta genes comprises the normal adult haemoglobin, haemoglobin A whereas 5 Hemoglobin A2, is composed of two alpha chains and two delta chains and constitutes up to 3% of total hemoglobin in adults. The hemoglobin with different chains varies in amino acids composition, their oxygen carrying capacity as well as in their stability. Embryonic hemoglobin (HbE) and HbF has better oxygen carrying capacity as compared to other hemoglobin types however the stability of HbE is lower as compared to adults Hbs. Parasites are totally dependent upon hemoglobin to replicate in the host. Therefore, it is of interest to study importance of Hb in RBC.

Malaria affects all age groups. However, the children are affected the most. According to WHO report there were an estimated 438 000 malaria deaths around the world in 2015, 69% of total deaths due to malaria are known to occur in children aged from 6 months to 5 years [1]. Children are considered to be most vulnerable groups in human population, Severe anaemia, hypoglycaemia and cerebral malaria are the features of severe malaria more commonly seen in children than in adults [13].

Though the parasite culture in RBC containing adult Hb is routinely performed [14] The studies on parasite culture in RBCs containing HbF by different groups show contradictory results. Some research have suggested that growth of Plasmodium is retarded in RBC containing HbF [15] while some suggests that under conditions of high oxygen the Plasmodium shows preference to RBC containing HBF with vigorous growth [16]. The inhibition of parasite growth in HbF has been due to factors present in mother's plasma [16].

In this study, we show the abundance of different amino acids in Plasmodium falciparum 3D7 and distribution of different amino acids according to its essential nature in host. We have also compared the amino acid composition of different chains of haemoglobin to determine the difference that leads to preference of RBC containing HbF, which might explain the disease severity in children.

## Methodology

The FASTA format of all protein sequence, 5,369 proteins, of Plasmodium was downloaded from PlasmoDB. Composition of protein sequences was completed using ProtParaman tool. ProtParaman tool is an online tool, which is freely available and determines the sequence composition and predicts other physical parameters [17]. Total amino acid composition was compiled using Microsoft Excel. The Charts and graphs were made using Microsoft excel. MSA of haemoglobin chains was performed using Clustal Omega tool from the EMBL-EBI Web Services [18].

## Results

### Abundance of Amino acids in parasite

**Figure 1:**
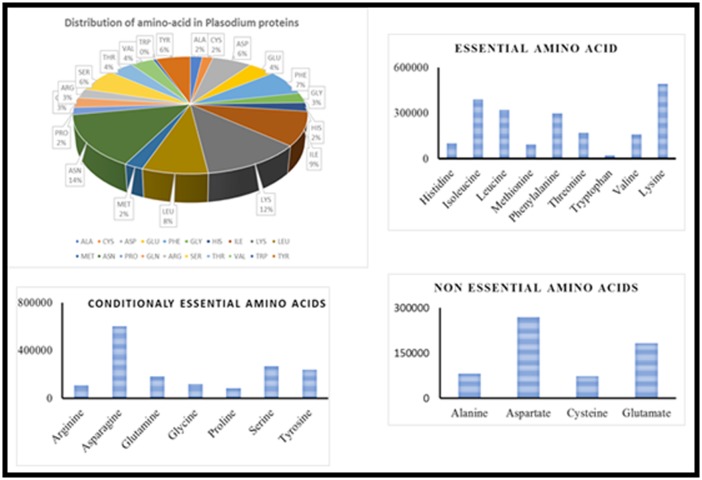
Abundance of amino-acid in total protein of Plasmodium. Sequences of all the 5639 proteins of Plasmodium were downloaded and abundance of amino acids were determined for individual protein by ProtParaman tool. The pie chart represents the abundance of the amino acids (A).The distribution of amino acids in Plasmodium proteins on the basis of essential (B), conditionally essential (C) and non-essential amino acids (D).

Complete protein sequences of 5369 were downloaded from PlasmoDB for Plasmodium falciparum 3D7. The amino acid compositions of total 5369 proteins are represented by pie chart (Figure 1A). As The amino-acid composition of Plasmodium falciparum proteins would suggest the co-relation between the amino-acids composition of haemoglobin and parasite proteins. The most incorporated amino acid in Plasmodium protein is N (13%), K (12%) and I (9%). We further analysed amino acids composition of Plasmodium protein according to the amino acid requirement by the host i.e. essential amino acids (Figure 1B), conditionally essential amino acids (Figure 1C) and non-essential amino acid (Figure 1D). It was observed that the essential and conditionally essential amino acids are widely incorporated in the Plasmodium proteins as compared to non-essential amino acids.

### Distribution of amino acids in Haemoglobin chains

The protein sequence of the haemoglobin chain was downloaded from NCBI and the composition of amino acid was determined using ProtParaman tool [17] as described above.

The fasta formats for different chain of the haemoglobins were obtained from NCBI. The assertion number of different chains of are as follows:

### NP_000550.2 hemoglobin subunit gamma:

MGHFTEEDKATITSLWGKVNVEDAGGETLGRLLVVYPWTQR FFDSFGNLSSASAIMGNPKVKAHGKKVLTSLGDATKHLDDLK GTFAQLSELHCDKLHVDPENFKLLGNVLVTVLAIHFGKEFTPE VQASWQKMVTAVASALSSRYH

### NP_005321.1 hemoglobin subunit epsilon

MVHFTAEEKAAVTSLWSKMNVEEAGGEALGRLLVVYPWTQ RFFDSFGNLSSPSAILGNPKVKAHGKKVLTSFGDAIKNMDNLK PAFAKLSELHCDKLHVDPENFKLLGNVMVIILATHFGKEFTPE VQAA WQKLVSAVAI ALAHKYH

### NP_000510.1 hemoglobin subunit delta [Homo sapiens]

MVHLTPEEKTAVNALWGKVNVDAVGGEALGRLLVVYPWTQ RFFESFGDLSSPDAVMGNPKVKAHGKKVLGAFSDGLAHLDNLKGTFSQLSELHCDKLHVDPENFRLLGNVLVCVLARNFGKEF TPQMQAAYQKVVAGVANALAHKYH

### NP_000509.1 hemoglobin subunit beta [Homo sapiens]

MVHLTPEEKSAVTALWGKVNVDEVGGEALGRLLVVYPWTQ RFFESFGDLSTPDAVMGNPKVKAHGKKVLGAFSDGLAHLDN LKGTFATLSELHCDKLHVDPENFRLLGNVLVCVLAHHFGKEF TPPVQAAYQKVVAGVANALAHKYH

### P69905.2 Hemoglobin subunit alpha

MVLSPADKTNVKAAWGKVGAHAGEYGAEALERMFLSFPTTK TYFPHFDLSHGSAQVKGHGKKVADALTNAVAHVDDMPNAL SALSDLHAHKLRVDPVNFKLLSHCLLVTLAAHLPAEFTPAVH ASLDKFLASVSTVLTSKY

### 3W4U_E|PDBID|CHAIN|SEQUENCE-zeta

MSLTKTERTIIVSMWAKISTQADTIGTETLERLFLSHPQTKTYFP HFDLHPGSAQLRAHGSKVVAAVGDAVKSIDDIGGALSKLSEL HAYILRVDPVNFKLLSHCLLVTLAARFPADFTAEAHAAWDKF LSVVSSVLTEKYR

Multiple sequence analysis of different chain of Hemoglobin was done using Clustal omega. The results show similarity between different chains of haemoglobin. Further, we looked at the distribution of amino acid in different haemoglobin chain (Table 1). We observe that the amino acid composition varies in different chains. None of the chains of Haemoglobin A (two alpha chains, and two beta chains) and Hemoglobin A2 (two alpha and two delta chains) contain I, an essential amino acid, in both alpha and delta chain. While Isoleucine is present in gamma chain haemoglobin F, (two gamma chains and two beta chains), Zeta and epsilon chain which forms part of HbE (Embryonic haemoglobin). This result show that I, which is an essential amino acid, comprises about 9% of total amino acids present in Plasmodium. Absence of this amino acid from Hb forces the parasite to rely upon exogenous supply of this amino acid, which may retard its growth.

## Discussion

Plasmodium parasite, scavenges the haemoglobin to meets its amino acid requirements while lipids are obtained from RBCs membrane. The distribution of amino acids in the proteins of Plasmodium shows incorporation of all the 20 amino acids. However, the amino acids, which are essential and conditionally essential to the host, are present in higher percentage than nonessential amino acids.

Among the essential amino acids leucine is required most followed by isoleucine. Among the conditionally essential amino acids N is required the most. N is present in parasite as repeat which might have role in immune evasion by antigenic variation. Other amino acids like arginine, which is essential for polyamine synthesis is important for robust growth of the parasite.

I make up to 9% of total amino acids in Plasmodium falciparum has to be obtained exogenously from blood, as it is absent in adult haemoglobin. Istvan et al. has shown that the absence of I affects parasite growth and I analogue inhibits the parasite growth [19]. The gamma chain of foetal haemoglobin (HbF) contains all the amino acids including I. Hence; the parasite residing in the foetal RBC does not require exogenous supply of isoleucine, which in turn might aid parasite to multiply exponentially. Study by Sauerzopf et.al., has shown that P. falciparum are equally permissive to growth in HbF and HbA in vitro further confirms that the growth of parasite will be aided by the presence of isoleucine. The percentage of HbF has been shown to be highest in foetus and 18% up to the age of 9 while in the age group of 10- 20 years its percentage falls to 8% [20, 21]. The presence of significant percentage of HbF up to the age of 9 might explain the venerability to malaria and death due to malaria.

## Conclusion

As Plasmodium solely depends upon the degradation of haemoglobin for its amino acid requirements it meets most of the nutritional requirements from haemoglobin. As HbF contains all the amino acids including isoleucine (absent in HbA) required by the parasite hence, this might be aid growth of parasite in HbF containing RBC. Besides at the age of 9 up to, 18% of RBC contains HbF. Hence, we hypothesise that presence of HbF would aid parasite growth leading to disease severity in children.

## Conflict of Interest

Authors declare no conflict of Interest

## Figures and Tables

**Table 1:** Dataset. The table lists the amino acid residues present in total protein of Plasmodium falciparum (A), essential amino acids (B), conditionally essential amino acid(C) and non-essential amino acid (D) residues present in Plasmodium falciparum.

(A) Amino Acid Residue	Number of Residue
ALA (A)	82537
CYS (C)	73987
ASP (D)	270290
GLU (E)	183037
PHE (F)	298756
GLY (G)	118471
HIS (H)	100949
ILE (I)	387902
LYS (K)	491170
LEU (L)	318735
MET (M)	91797
ASN (N)	600456
PRO (P)	83223
GLN (Q)	115618
ARG (R)	110769
SER (S)	267090
THR (T)	171384
VAL (V)	159380
TRP (W)	20830
TYR (Y)	238546
	
(B) Essential amino acid	Number of residues
Histidine (H)	100949
Isoleucine (I)	387902
Leucine (L)	318735
Methionine(M)	91797
Phenylalanine (F)	298756
Threonine (T)	171384
Tryptophan (W)	20830
Valine(V)	159380
Lysine (K)	491170
	
(C) Conditionally essential amino acid	Number of residues
Arginine (R)	110769
Asparagine (N)	600456
Glutamine (Q)	183037
Glycine (G)	118471
Proline (P)	83223
Serine (S)	267090
Tyrosine (Y)	238546
	
(D) Non-essential amino acid	Number of residues
Alanine (A)	82537
Aspartate (D)	270290
Cysteine (C)	73987
Glutamate (E)	183037

**Table 2:** Table showing the distribution of different amino acids in different chains of haemoglobin.

Hb SUBUNIT	A	C	D	E	F	G	H	I	K	L	M	N	P	Q	R	S	T	V	W	Y	
GAMMA	12	1	8	8	8	12	7	3	12	17	3	5	4	4	3	11	11	13	3	2	147
EPSILON	17	1	5	9	9	9	7	5	14	16	4	7	6	3	2	9	6	13	3	2	147
DELTA	15	2	7	7	8	13	7	0	11	18	3	8	6	5	4	6	5	17	2	3	147
BETA	15	2	7	8	8	13	9	0	11	18	2	6	7	3	3	5	7	18	2	3	147
ALPHA	21	1	8	4	7	7	10	0	11	18	3	4	7	1	2	11	9	13	1	3	141
ZETA	16	1	8	6	7	6	7	7	9	17	2	1	5	3	6	13	12	11	2	3	142

**Figure 2:**
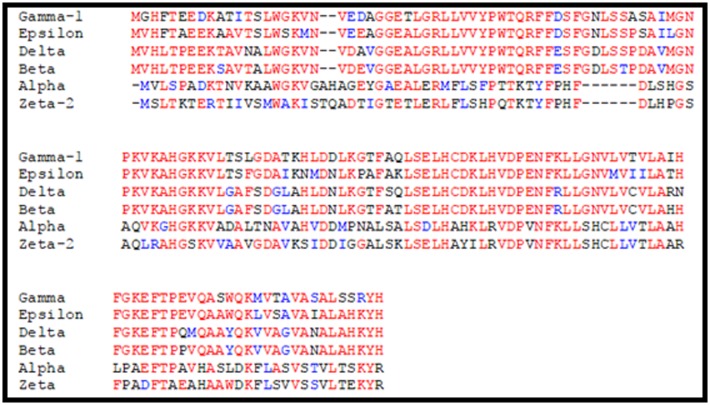
Multiple sequence alignment of amino acid sequence of different chains of hemoglobin gamma (NP_000550.2) Epsilon (NP_005321.1), Delta (NP_000510.1) Beta (NP_000509.1), Alpha (P69905.2), and Zeta (3W4U:E). Identical and similar amino acids are highlighted with similar font colors.
